# Development and Validation of the Isotretinoin Hesitancy Scale for Acne Vulgaris: A Preliminary Study

**DOI:** 10.1111/jocd.16589

**Published:** 2024-09-18

**Authors:** Esra Agaoglu, Imran Gokcen Yılmaz Karaman, Cennet Yastıbas Kacar, Hilal Kaya Erdogan, Talha Mutlu, Ayse Serap Karadag

**Affiliations:** ^1^ Department of Dermatology, Faculty of Medicine Eskisehir Osmangazi University Eskisehir Turkey; ^2^ Department of Psychiatry, Faculty of Medicine Eskisehir Osmangazi University Eskisehir Turkey; ^3^ Department of Psychology, Faculty of Humanities and Social Science Adana Alparslan Turkes Science and Technology University Adana Turkey; ^4^ Department of Dermatology Istanbul Arel University Medical Faculty Istanbul Turkey

**Keywords:** acne vulgaris, hesitancy, isotretinoin, scale, treatment

## Abstract

**Background:**

Oral isotretinoin is the most effective systemic treatment for acne patients who fail to respond to other forms of therapies. However, hesitations and concerns regarding its side effect profile may detain the patients from treatment. This study aimed to develop and validate the Isotretinoin Hesitancy Scale (IHS) among acne patients.

**Methods:**

A cross‐sectional study was conducted with 100 acne patients who had not used isotretinoin previously. A 22‐item scale was created based on the related literature and expert opinions. The items of the scale related to beliefs and worries about isotretinoin were formatted with response options: agree, indecisive, and disagree. In this study, construct validity was tested with exploratory factor analysis, and reliability was tested with internal consistency and split‐half reliability.

**Results:**

The results of exploratory factor analysis indicated a three‐factor solution with a total of 14 items, explaining 57% of the total variance. The first factor (Hesitancy Related to Reversible Adverse Effects: 6 items) accounted for 30% of the variance, the second factor (Hesitancy Related to Irreversible Adverse Effects: 4 items) accounted for 16% of the variance and the third factor (Isotretinoin‐related Anxiety: 4 items) accounted 11% of the variance. The internal consistency of the three factors was calculated as 0.79, 0.78, and 0.72, respectively. The Cronbach's alpha score of the total scale was found to be 0.81, and split‐half reliability was found to be 0.87.

**Conclusions:**

The IHS is the first scale that provides a valid and reliable assessment of isotretinoin hesitancy in acne patients. Eliminating isotretinoin hesitancy may reduce acne‐related clinical and psychosocial consequences.

## 1 Introduction

Acne vulgaris is a chronic inflammatory disease with a worldwide prevalence. It is the most common skin disease and affects approximately 80% of adolescents and young adults [1]. Acne frequently affects the face, and scar formation is a permanent complication seen in approximately 95% of the patients. That leads to long‐term psychosocial effects, negatively affecting the patient's quality of life. The negative impact of both acne and acne scarring on psychological health may lead to social isolation, lower levels of self‐esteem, depression, and anxiety regardless of disease severity [2, 3].

Isotretinoin (13‐cis retinoic acid), a derivative of retinol, was approved for treating acne vulgaris in 1982, being the only drug that acts on all processes of acne pathogenesis. Current opinion is recommending isotretinoin even in patients with mild to moderate acne who are unresponsive to other treatments that cause scars or psychological stress. It is well known that isotretinoin treatment is associated with several dose‐dependent and tolerable mucocutaneous, ocular, and musculoskeletal adverse effects [4, 5].

Contrary to its effectiveness, there is an isotretinoin‐treatment gap in patients with acne. Hesitations and concerns in acne patients regarding its side effect profile may detain the use of isotretinoin treatment [6, 7]. The present study aims to develop and validate the Isotretinoin Hesitancy Scale (IHS) among patients with acne vulgaris.

## 2 Materials and Methods

### 2.1 Study Design

The present study has a cross‐sectional design. The researchers submitted the study scale to the patients who applied to the Dermatology Outpatients Clinic of Eskısehir Osmangazi University Faculty of Medicine. One hundred patients were recruited. Inclusion criteria were patients diagnosed with acne, being 16 years of age and older, and not having received systemic isotretinoin previously. All patients included in the study and the parents of patients under 18 were asked to fill out an informed consent form before the scale. The local ethics committee approved the study protocol (approval number: 48, date: February 21, 2023).

### 2.2 Measurements

#### 2.2.1 Sociodemographic and Clinical Characteristics Form

In the first part of the survey, sociodemographic characteristics, age of acne onset, duration of acne, severity of acne, acne type, and location were recorded by a dermatologist. Acne severity scores were evaluated using the Investigator's Global Assessment (IGA). According to the IGA, patients with a few scattered comedones were evaluated in grade 1, patients with some non‐inflammatory lesions and few inflammatory lesions were evaluated in grade 2, patients with multiple inflammatory lesions evident with several to many papules/pustules were evaluated in grade 3 and many comedones and papules/pustules alongside a few nodulocystic lesions were evaluated in grade 4.

Patients were asked whether they had received any treatment for acne before and which medications they used. The patients were also asked whether they were informed about isotretinoin and had any concerns about it. The responses to the questions were “yes” and “no.”

#### 2.2.2 Visual Analog Scale (VAS)

The patients' anxiety level regarding isotretinoin treatment was evaluated with the VAS. VAS is a single‐item measurement that is commonly used in healthcare settings to measure anxiety related to medical treatments. The scale value is between 0 and 10 [8].

#### 2.2.3 The Isotretinoin Hesitancy Scale (IHS)

While developing the scale, an item pool was initially determined, collaborating with specialists, including three dermatologists and one psychiatrist. The items of the scale were developed based on the studies examining the concerns of acne patients regarding systemic isotretinoin [6, 7, 9, 10, 11, 12] and studies on topical steroid phobia [13] and atopic dermatitis [14].

**FIGURE 1:**
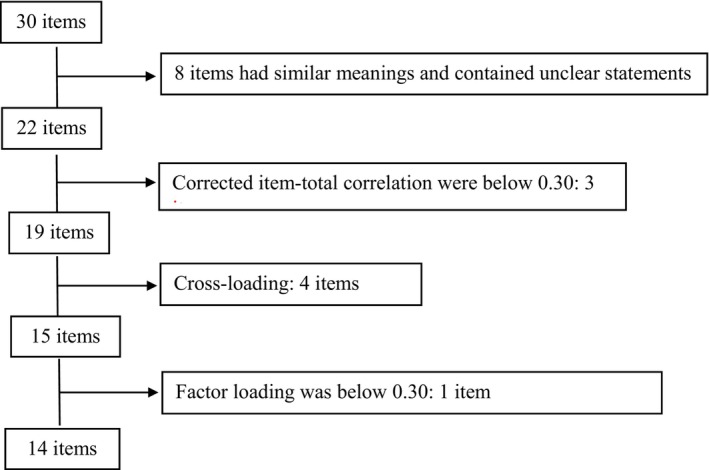
Selection procedure of items (flow chart).

One dermatologist consulted the generated items in the field of acne and one psychiatrist in the field of psychodermatology, and their suggestions were taken. Regarding the beliefs and worries about the isotretinoin treatment, 30 items were obtained (Figure 1). Before the data collection, all items were checked by the researchers in terms of clarity and acceptability. Therefore, the eight items were removed from the scale for having similar meanings, measuring the facts about treatments that are not the study's objective, and containing unclear statements. The final version of the scale, which consists of 22 items, was applied to the participants. The participants were asked to rate the statements in the scale on the 3‐point scale format with the following response options: agree, indecisive, and disagree. The total scores ranged from 14 to 42. Higher scores from IHS referred to higher hesitancy to isotretinoin treatment.

### 2.3 Statistical Analysis

This cross‐sectional study was conducted with a paper‐pencil survey method. Data analyses were carried out using SPSS (Windows) Version 23. Descriptive statistics were examined as frequency, mean, and standard deviation. There is no missing value. According to the examination of normality assumptions, the data were considered to be normally distributed. We calculated the Cronbach's alpha score, corrected item‐total correlation, and split‐half reliability for the scale's reliability. Regarding construct validity, we performed exploratory factor analysis (EFA) using the principal component analysis extraction method with both promax and direct oblimin rotation to decide the factorial structure of the scale. To obtain a clear construct, we decided to utilize promax rotation. Before conducting EFA, we assessed the Kaiser–Meyer–Olkin (KMO) and Barlett's sphericity tests for the data's properties. For group differences regarding sex and marital status, we employed an independent sample *t*‐test and one‐way ANOVA with Bonferonni correction; for continuous demographic variables such as age, we conducted Pearson correlation analysis. All statistical analyses are considered as significant *p* < 0.05.

## 3 Results

### 3.1 Demographics and Clinical Characteristics of the Sample

One hundred acne patients were recruited for the preliminary analyses of the scale. The mean age of the patients was 22.72 ± 4.12. The mean age of acne onset was 17.46 ± 3.73 years. Adolescent (77.0%) and comedonal (59.0%) acne were the most frequent types of acne vulgaris. Forty‐five (45.0%) of the patients were previously treated with topical agents, while 21 (21.0%) of the patients received topical agents and oral antibiotics (Table 1).

**TABLE 1:** Sociodemographic and clinical characteristics of the patients.

Mean age ± SD		22.72 ± 4.12
Sex	Female	72 (72.0%)
	Male	28 (28.0%)
Marital status	Single	90 (90.0%)
	Married	10 (10.0%)
Family history of acne vulgaris	Present	35 (35.0%)
	Absent	65 (65.0%)
Mean age of acne onset ± SD		17.46 ± 3.73
Mean duration of disease (months) ± SD		53.83 ± 37.50
Acne severity^a^	1	6 (6.0%)
	2	40 (40.0%)
	3	36 (36.0%)
	4	18 (18.0%)
Type of acne	Adolescence	77 (77.0%)
	Postadolescence	19 (19.0%)
	Comedonal	59 (59.0%)
	Papulopustular	52 (52.0%)
	Nodulocystic	42 (42.0%)
Previous treatments	Topical agent	45 (45.0%)
	Oral antibiotic	4 (4.0%)
	Topical agent + oral antibiotic	21 (21.0)

Abbreviation: SD, Standard deviation.

^a^ Disease severity according to the Food and Drug Administration criteria.

Seventy‐nine (79.0%) of the patients stated that a dermatologist had not previously recommended isotretinoin. When the patients' concerns were questioned, 51 (51.0%) of the patients stated that they did not have detailed information about isotretinoin. Fifty‐four (54.0%) patients expressed concerns about isotretinoin treatment (Table 2). The mean VAS score of the patients was 3.67 ± 3.10.

**TABLE 2:** Answers of the patients to the questions regarding isotretinoin treatment.

Questions	*n* (%)
Have you ever been recommended isotretinoin treatment for your acne by a dermatologist?	
Yes	21 (21.0%)
No	79 (79.0%)
Do you have enough information about isotretinoin treatment for acne?	
Yes	49 (49.0%)
No	51 (51.0%)
Do you have concerns about isotretinoin treatment?	
Yes	54 (54.0%)
No	46 (46.0%)

### 3.2 Item Analysis

The initial analysis examined the frequency of the responses of the items and descriptive statistics. No floor or ceiling effects were observed. The skewness and kurtosis scores of the items ranged between acceptable scores. The descriptive statistics, including mean, standard deviation, skewness, and kurtosis of the scale, were presented in Table 3.

**TABLE 3:** The descriptive statistics of the scale.

Items	Mean	Std. Deviation	Skewness	Kurtosis	Corrected item‐Total correlation
Isotretinoin treatment can lead to dryness of lips, nose, and eyes.	2.80	0.426	−1.91	2.75	0.368
Isotretinoin treatment may cause damage to the liver.	2.65	0.500	−0.884	−0.639	0.459
Isotretinoin treatment may cause elevation of cholesterol level.	2.32	0.583	−0.191	−0.608	0.518
In the case of pregnancy, isotretinoin treatment may cause congenital defects in the baby.	2.14	0.635	−0.124	−0.535	0.416
Isotretinoin treatment may cause depression.	2.35	0.687	−0.584	−0.741	0.493
Isotretinoin treatment may prevent height gain.	1.83	0.603	0.089	−0.354	0.342
Isotretinoin treatment may cause infertility in women.	1.90	0.627	0.075	−0.440	0.400
Isotretinoin treatment may cause infertility in men.	1.82	0.609	0.111	−0.408	0.413
Isotretinoin treatment may have many side effects.	2.67	0.551	−1.45	1.19	0.532
Side effects of isotretinoin treatment may affect my daily life.	2.61	0.529	−0.870	−0.404	0.463
I am afraid of using isotretinoin for a long period.	2.40	0.752	−0.814	−0.764	0.377
I will wait as long as I can before using isotretinoin treatment.	2.29	0.782	−0.563	−1.14	0.451
I stop the isotretinoin treatment as soon as possible.	2.35	0.729	−0.654	−0.852	0.465
I need more reassurance about isotretinoin treatment.	2.51	0.674	−1.04	−0.107	0.438

The corrected item‐total correlation was calculated to determine whether each item contributed significantly to the scale. The Cronbach's alpha scores of the three items were below 0.30. Therefore, these three items were removed from the scale. We continued the factor analysis with the 19 remaining items of the scale. We used principal component analysis as an extraction method with promax rotation and direct oblimin to decide the number of factors. We hypothesized a two‐factor model; however, a three‐factor structure has emerged in factor analysis. The emerging three‐factor solution presented a more precise and interpretable factorial structure than the other solution. Regarding the promax and direct oblimin rotation, we detected some cross‐loading items. To achieve a clearer factor solution and theoretical framework, we decided to use Promax rotation. Factor analyses, using Principal Component Analysis with Promax rotation, were conducted, and four items were deleted due to the cross‐loading. The remaining 15 items were again tested to understand the three‐factor solution. One of the items was also dropped from the scale since this item had a lower factor loading (< 0.30). After dropping, the three‐factor solution explained the most significant amount of the variance. The final version of the scale contained 14 items, and the obtained three‐factor solution could explain 57% of the total variance. The first factor had six items labeled as Hesitancy Related to Reversible Adverse Effects, accounting for 30% of the variance; four items loaded onto the second factor labeled as Hesitancy Related to Irreversible Adverse Effects, accounting for 16% of the variance; and the following four items loaded onto the third factor labeled as Isotretinoin‐related Anxiety, accounting for 11% of the variance (see Table 4). To determine whether the data were appropriate for exploratory factor analysis, Bartlett's test of sphericity and the KMO for sampling adequacy were examined. Bartlett's test of sphericity was significant (*p* < 0.001), and the KMO score was 0.68.

**TABLE 4:** Exploratory factor analysis results of the scale.

	Factor 1	Factor 2	Factor 3
Isotretinoin treatment can lead to dryness of lips, nose, and eyes.	0.842		
Isotretinoin treatment may have many side effects.	0.783		
Isotretinoin treatment may cause damage to the liver.	0.729		
Side effects of isotretinoin treatment may affect my daily life.	0.689		
Isotretinoin treatment may cause depression.	0.635		
Isotretinoin treatment may cause elevation of cholesterol level.	0.516		
Isotretinoin treatment may cause infertility in men.		0.945	
Isotretinoin treatment may cause infertility in women.		0.857	
Isotretinoin treatment may prevent height gain.		0.778	
In the case of pregnancy, isotretinoin treatment may cause congenital defect in the baby.		0.425	
I am afraid of using isotretinoin for a long period.			0.890
I stop the isotretinoin treatment as soon as possible.			0.724
I will wait as long as I can before using isotretinoin treatment.			0.711
I need more reassurance about isotretinoin treatment.			0.569

### 3.3 Reliability Analysis

The Cronbach's alpha score of the final form of the scale was found to be 0.81, the internal consistency of the first factor (Hesitancy Related to Reversible Adverse Effects) was calculated as 0.79, the second factor (Hesitancy Related to Irreversible Adverse Effects) was calculated as 0.78, and the final factor (Isotretinoin‐related Anxiety) was found to be 0.72. The corrected item‐total correlation ranged from 0.52 to 0.34. Each item was significantly correlated with the total scores of the scale (*p* < 0.001). We also tested split‐half reliability, and it was found to be 0.87.

### 3.4 Group Differences of Total Score and Subscale Scores of the IHS

We examined socio‐demographic differences and correlates for the total and subscales of the IHS. Regarding group differences, men and women did not significantly differ on the total and the subscales of the IHS (*p* > 0.05). Marital status, education level, having a psychiatric disorder, and having a systemic disorders also did not differed for the total and sucbscales of the IHS (*p* > 0.05). However, IHS total score and Hesitancy Related to Irreversible Adverse Effects subscale score were positively correlated to the participants' age (respectively *r* = 0.214, *p* = 0.033; *r* = 0.257, *p* = 0.010). Hesitancy Related to Reversible Adverse Effects and Isotretinoin‐related Anxiety subscale scores were not associated with the participants' age (*p* > 0.05).

Furthermore, VAS scores (give a number between 0 and 10 for the severity of your hesitation about isotretinoin) were associated with IHS total scores (*r* = 0.477, *p* < 0.001), Hesitancy Related to Reversible Adverse Effects subscale scores (*r* = 0.439, *p* < 0.001), Hesitancy Related to Irreversible Adverse Effects subscale scores (*r* = 0.206, *p* = 0.040), and Isotretinoin‐related Anxiety subscale scores (*r* = 0.385, *p* < 0.001).

## 4 Discussion

This study aimed to develop and validate the IHS. To the best of our knowledge, no assessment tool evaluates the hesitations that make acne patients anxious about isotretinoin treatment. The present study demonstrated that IHS is valid and reliable among Turkish patients with acne vulgaris.

The IHS, consisting of 14 items, has a 3‐point scale format with the following response options: agree, indecisive, and disagree, and the scores obtained from the scale ranged from 14 to 42. The EFA results exhibited a three‐factor solution explaining 57% of the total variance. Three factors were Hesitancy Related to Reversible Adverse Effects, Hesitancy Related to Irreversible Adverse Effects, and Isotretinoin‐related Anxiety. The internal consistency of the total scores of the IHS was found to be good; the subscales of the IHS refer to reliable values. Furthermore, the split‐half reliability result was also found to be good reliability. Based on our results, IHS promised a reliable and valid measurement tool for the Turkish population.

Isotretinoin hesitancy is a common issue in acne patients and causes resistance to treatment. When discussing isotretinoin with acne patients, many of them have prejudices about the drug. It is well documented that the most common concerns about initiating isotretinoin treatment are its side effect profile and the necessity of long‐term daily use [6, 7, 9, 10, 11, 12]. Most side effects are dose‐dependent and can be easily managed using lower dosages [5, 15]. Considering the impact of acne scars on psychological and economic burdens, it is recommended to start isotretinoin without delay in mild to moderate acne that causes scarring [3]. Furthermore, early intervention of acne scars with isotretinoin could decrease the costs spent on cosmetic procedures [2, 16].

Items of Hesitancy Related to Reversible Adverse Effects are mainly comprised of dry lips, nose, and eyes, liver injury, risk of depression, elevation of cholesterol levels, and side effects affecting daily life. Many studies reported in the literature have shown mucocutaneous side effects are the most common side effects of isotretinoin [6, 7, 9, 11]. These side effects are dose‐dependent and predictable; they can easily be managed with topical moisturizers and ocular lubricants. To minimize those side effects, the clinician informs patients before the treatment, and regular use of moisturizers is recommended from the first days of isotretinoin [15, 17].

Laboratory alterations comprise about 2% of side effects during isotretinoin, such as an increase in blood level of triglycerides (44%), total and LDL cholesterol (33%), and transaminases (11%). These reversible side effects do not require treatment discontinuation. Before the isotretinoin, the clinician generally performs laboratory monitoring of serum lipids and liver transaminases. Therefore, long‐term use of isotretinoin is not recommended in patients with underlying high blood levels of lipid and transaminase [5, 17]. Many studies on isotretinoin have shown that acne patients have a good awareness of the elevation of lipid profile [10, 11, 12]. The importance of monitoring triglyceride levels during isotretinoin therapy is related to the concern about pancreatitis due to hypertriglyceridemia [17]. However, elevation in triglyceride levels resulting from isotretinoin is mild (< 300 mg/dL or 3.4 mmol/L) and can be managed with alteration of lifestyle or dietary regime without cessation of treatment [18]. Our study revealed that an elevation in serum lipid levels was such a side effect that caused patients to hesitate about treatment.

The mild elevation in transaminases during treatment indicates that isotretinoin is not a severe cause of liver injury. The use of other medications or dietary supplements may also increase liver transaminases. Additionally, a temporary elevation of liver transaminases due to isotretinoin does not lead to liver damage [5]. In our patients, the concern of the possibility of liver damage was among the side effects that caused hesitation about isotretinoin.

The relationship between isotretinoin and psychiatric side effects is based mainly on case series [19, 20]. Isotretinoin, with its lipophilic structure, crosses the blood–brain barrier and affects the components of the serotoninergic system, leading to decreased serotonin signaling [21]. It is suggested that the impairment of serotonin signaling may cause depression in susceptible individuals [22]. However, meta‐analyses do not support an association between isotretinoin and depression [23, 24]. On the other hand, even mild forms of acne can increase the risk of depression, and successful treatment of acne with isotretinoin may improve psychological status [15, 24]. Although few reported cases of depression resolved with discontinuation of isotretinoin [19, 20], it is mandatory to question whether patients have depressive symptoms before and during treatment. The use of isotretinoin is not recommended in patients with currently depressive or psychotic symptoms [5, 24]. The selected item regarding the concern that isotretinoin causes depression is consistent with those previously mentioned by some authors [7, 10].

Looking at the items of Hesitancy Related to Irreversible Adverse Effects, both our male and female patients had concerns about future fertility. Contrary to the patient's concerns, isotretinoin positively affects male fertility [25, 26]. Cinar et al. [25] showed the positive effect of low‐dose isotretinoin on spermiogram parameters. Amory et al. [26] found an improvement with isotretinoin in sperm concentration and morphology in male patients with oligo‐azoospermia. In females, Sikar Aktürk et al. [27] evaluated anti‐Mullerian hormone (AMH) levels before and after treatment with isotretinoin compared to controls. They observed no significant difference between post‐treatment AMH levels and AMH levels of the control group. Additionally, in a study of females, a temporary decrease was observed in AMH, antral follicle count (AFC), and ovarian volume (OV) parameters after 6 months of isotretinoin [28]; however, it has been shown that these changes return to normal 12 months after treatment [29]. Therefore, there is insufficient evidence that isotretinoin negatively affects fertility in female patients with acne.

The teratogenic effect of isotretinoin is the most serious concern, which is considered a dose‐independent and irreversible adverse event in women of childbearing age. Therefore, it is mandatory to exclude the possibility of pregnancy before isotretinoin treatment. Also, female patients are advised to use two reliable contraceptive methods during and up to one month after treatment [16]. Patients' awareness of the teratogenicity of isotretinoin and their compliance with precautions have been demonstrated in many studies [7, 10, 11, 12]. Similarly, an item related to the teratogenicity of isotretinoin is a well‐known irreversible side effect in our patients.

Premature epiphyseal closure and the resulting decrease in height growth are significant side effects that may develop secondary to prolonged administration and high‐dose usage of oral isotretinoin. In clinical practice, acne patients are generally screened for mucocutaneous side effects and laboratory evaluations, but there is no screening to evaluate the decrease in height growth, especially in pediatric patients. Additionally, arthralgia, a common side effect of isotretinoin, may mask knee pain caused by epiphyseal closure [5]. Although few cases of premature epiphyseal closure have been reported in acne patients [30, 31, 32], it is not recommended to use high doses of isotretinoin in the pediatric population [33]. This concern about height growth regarding isotretinoin was documented for the first time in this study by our patients.

According to the Isotretinoin‐related Anxiety items, patients were reluctant to receive treatment for an extended period and perceived isotretinoin as the last treatment option. They also stated that they tended to complete isotretinoin treatment quickly if they started. Even though our patients had not previously used isotretinoin for acne, there are many reasons for this anxiety, such as misinformation from friends or family and the internet.

The present study has several limitations. We employed a small sample size from a single center. Besides, we could not conduct confirmatory factor analysis (CFA). Thus, further studies should recruit a large sample from multiple centers and replicate the scale by conducting both EFA and CFA.

## 5 Conclusion

The IHS may help clinicians identify hesitations and concerns that prevent acne patients from initiating treatment. Evaluating perceptions or myths regarding isotretinoin may help clinicians better understand the patients' concerns. It is essential to devote sufficient time and attention to the educational aspect of treatment. Correctly informing patients by the clinicians is crucial to reducing prejudices against isotretinoin. Increasing treatment adherence will reduce both clinical and psychosocial adverse outcomes of acne. The IHS will be functional in future studies to evaluate levels of isotretinoin hesitancy and how the concerns of acne patients can change about isotretinoin treatment when given proper education.

## Ethics Statement

The local ethics committee approved the study protocol (approval number: 48, date: February 21, 2023).

## Conflicts of Interest

The authors declare no conflicts of interest.

## Data Availability

Data available on request from the authors.
